# Supplementary figures and images for: Compartment specific responses to contractility in the small intestinal epithelium
Source: PLoS Genet. 2024 Mar 22;20(3):e1010899. doi: 10.1371/journal.pgen.1010899 (PMC10990186; doi:10.1371/journal.pgen.1010899)

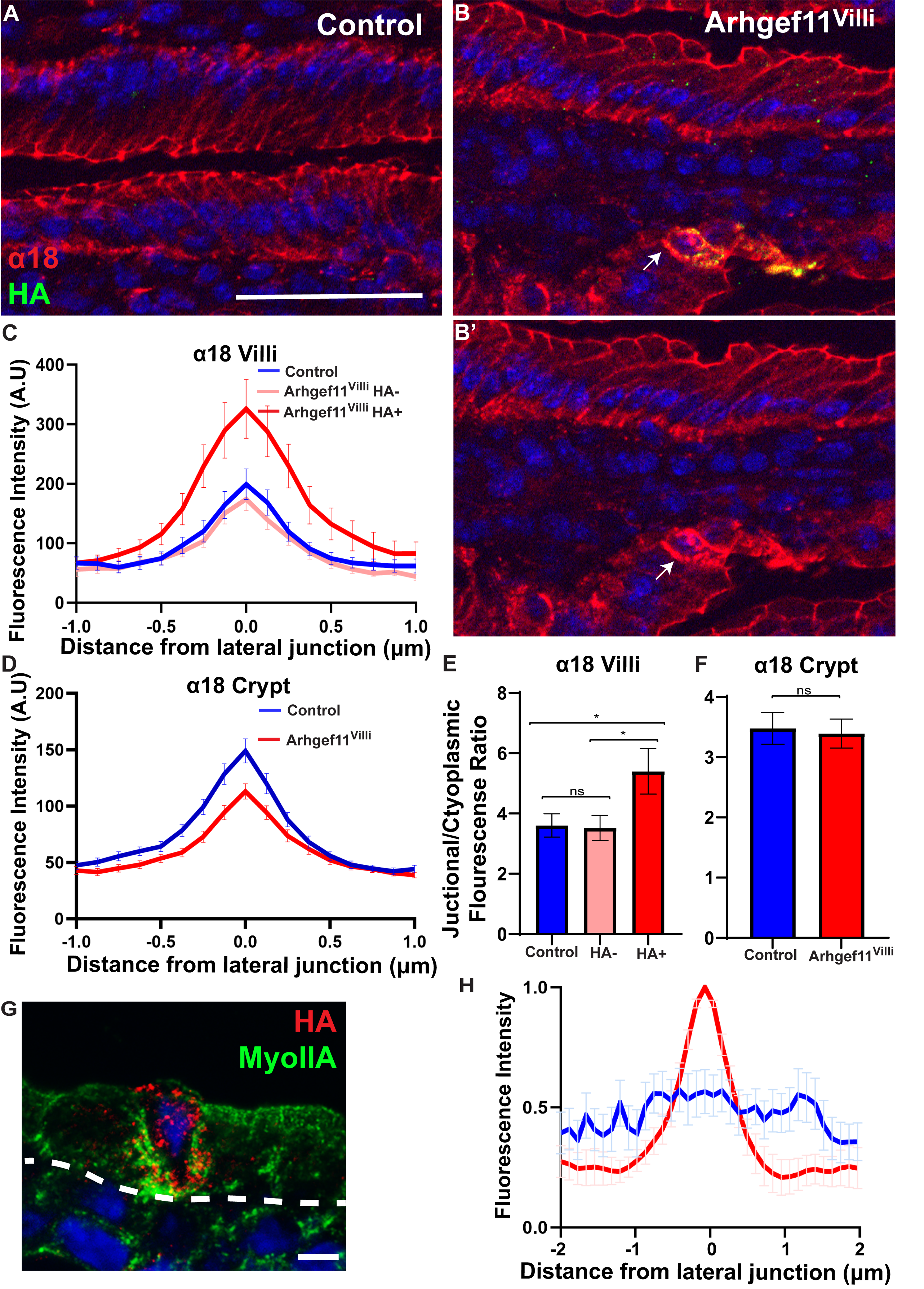

Supplement: S1 Fig — (A and B) Immunofluorescence staining on small intestine sections of α-catenin α18 (red) and HA (green). Note that α18 staining is highest in HA+ cell in B’. Scale bar 50μm. (C and D) Quantification of lateral junction α18 intensity in (C) villar and (D) crypt cells. Data are mean ±SEM. (E and F) Average ratio of the junctional and cytoplasmic fluorescence values in (E) villi, p = 0.0254, ordinary one-way ANOVA, n = 24 cells for control and n = 20 for HA- cells and n = 18 HA+ cells for Arhgef11Villi and (F) crypts p = 0.809, unpaired t-test, n = 48 cells for control and n = 47 for Arhgef11Villi from 3 mice per genotype. (G) Immunofluorescence staining of MyoIIA (green) and HA (red) in Arhgef11Villi villar intestinal epithelia. Scale bar 15μm. (H) Quantification of lateral junction MyoIIA intensity. Data are mean ±SEM. (TIF) [file pgen.1010899.s001.tif]

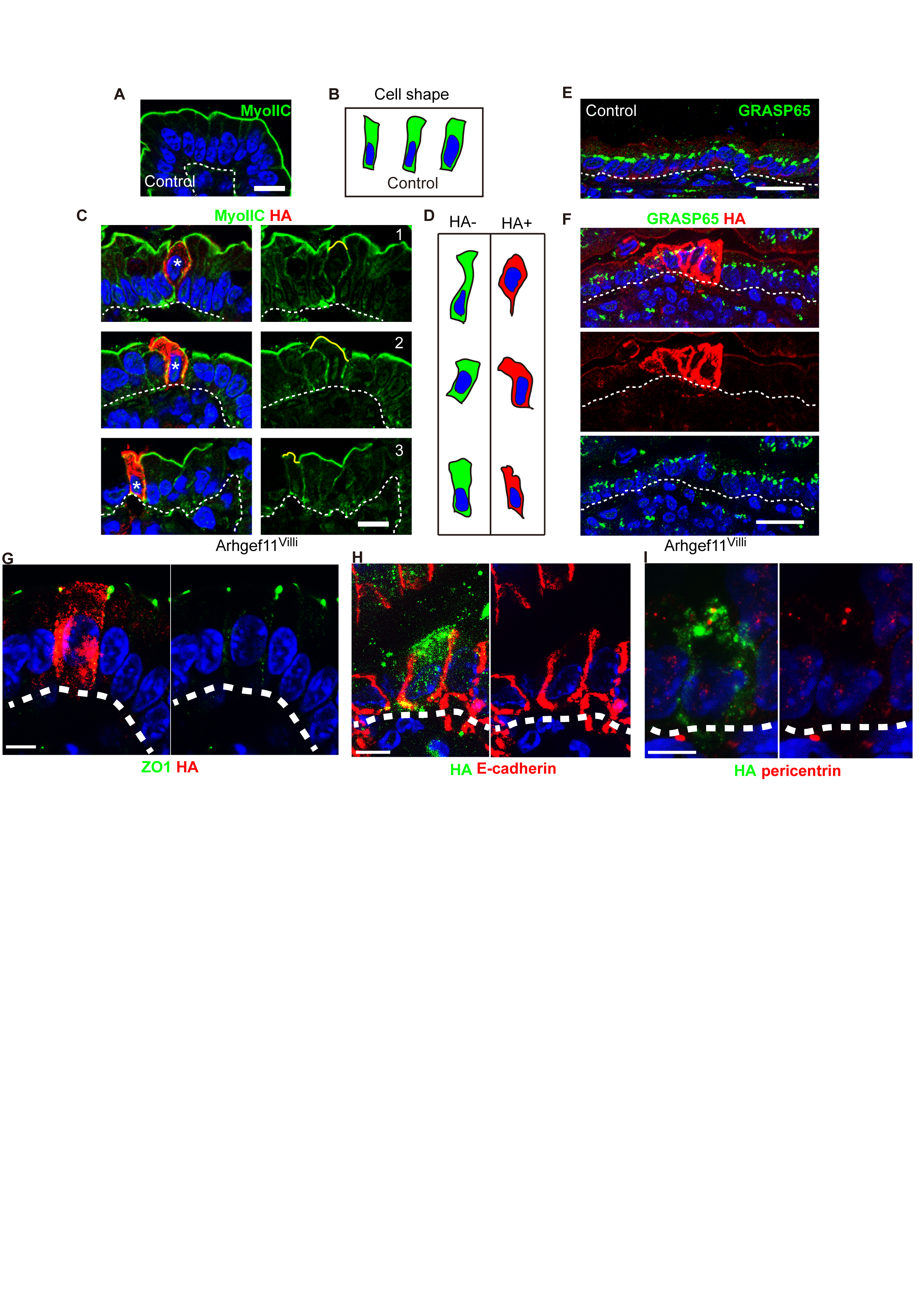

Supplement: S2 Fig — (A and C) Immunofluorescence staining of MyoIIC (green) and HA (red) in control (A) and Arhgef11Villi (C) villar intestinal epithelia. Scale bars for A and C, 20 μm. (B and D) Traces of representative villar epithelial cell shapes in control (B) and Arhgef11Villi (D) HA-(green) and HA+(red) sections. (E and F) Immunofluorescence staining of GRASP65 (green) and HA (red) in control (E) and Arhgef11Vill (F) epithelial sections. Dotted lines mark the basement membrane. Scale bar for E and F, 40 μm. (G) Immunofluorescence staining of ZO1 (green) and HA (red) in Arhgef11Villi villar intestinal epithelia. (H) Staining of HA (green) and E-cadherin (red) in Arhgef11Villi villar intestinal epithelia. (I) Staining of HA (green) and pericentrin (red) in Arhgef11Villi villar intestinal epithelia. Scale bar for G-I 15μm. Dotted lines mark the basement membrane. (TIF) [file pgen.1010899.s002.tif]

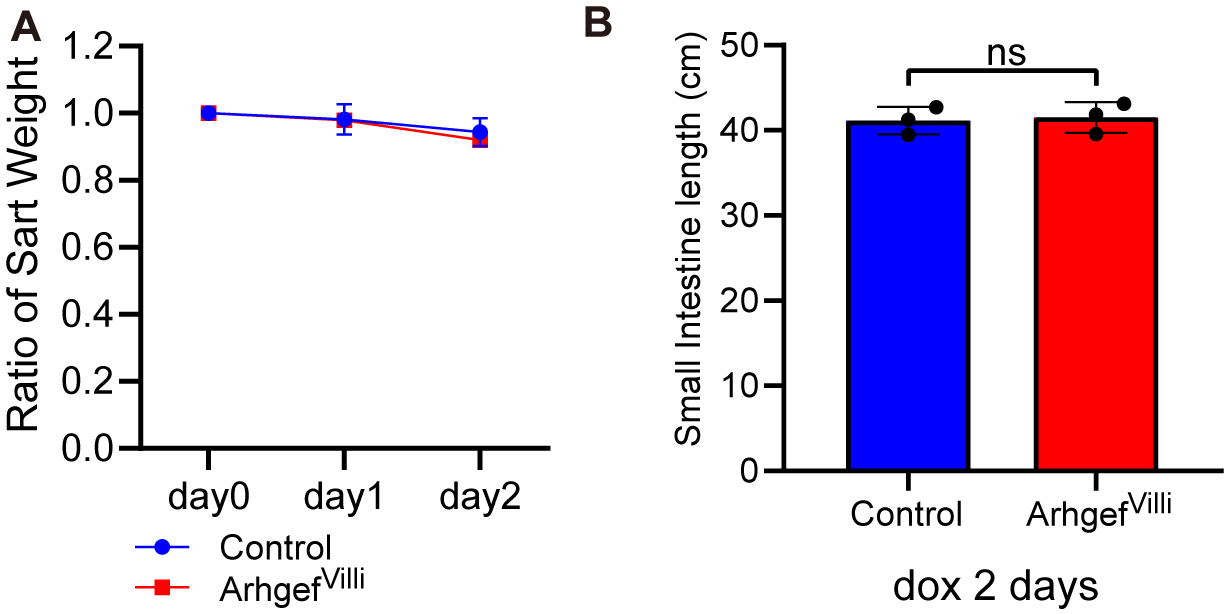

Supplement: S3 Fig — (A) Ratio of weight change after one and two days of doxycycline induction for control and Arhgef11Villi, n = 3 animals for each genotype. (B) Quantification of small intestine length of control and Arhgef11Villi animals in cm. (TIF) [file pgen.1010899.s003.tif]

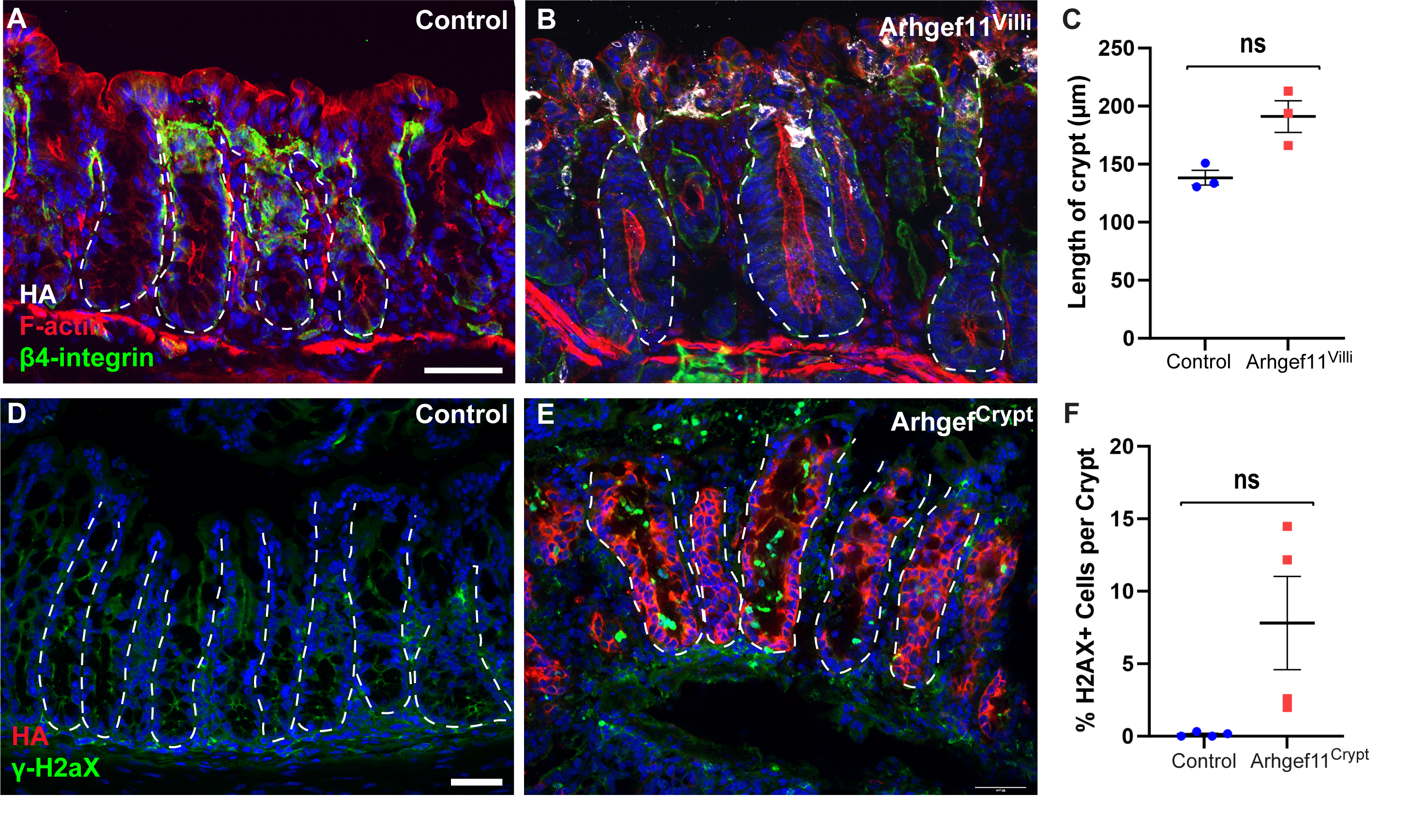

Supplement: S4 Fig — (A-B) Immunofluorescence images of (A) control and (B) ArhgefVilli large intestine sections stained with F-actin (green), β4-integrin (red), and HA (white). Dashed lines denote individual crypts. Scale bar 50μm. (C) Quantification of colon crypt length in microns. For control n = 62 crypts from 3 animals. For ArhgefVilli, n = 52 crypts from 3 animals. p = 0.1166, paired t-test. (D-E) Immunofluorescence images of (D) control and (E) ArhgefCrypt large intestine sections stained with γ-H2aX (green) and HA (red). Dashed lines denote individual crypts. Scale bar 50μm. (F) Quantification of percentages of γ-H2aX positive crypt cells after 12 hours of dox induction n = 4 animals (44 crypts) for control and 4 animals (68 crypts) ArhgefCrypt. p = 0.0961, paired t-test. (TIF) [file pgen.1010899.s004.tif]

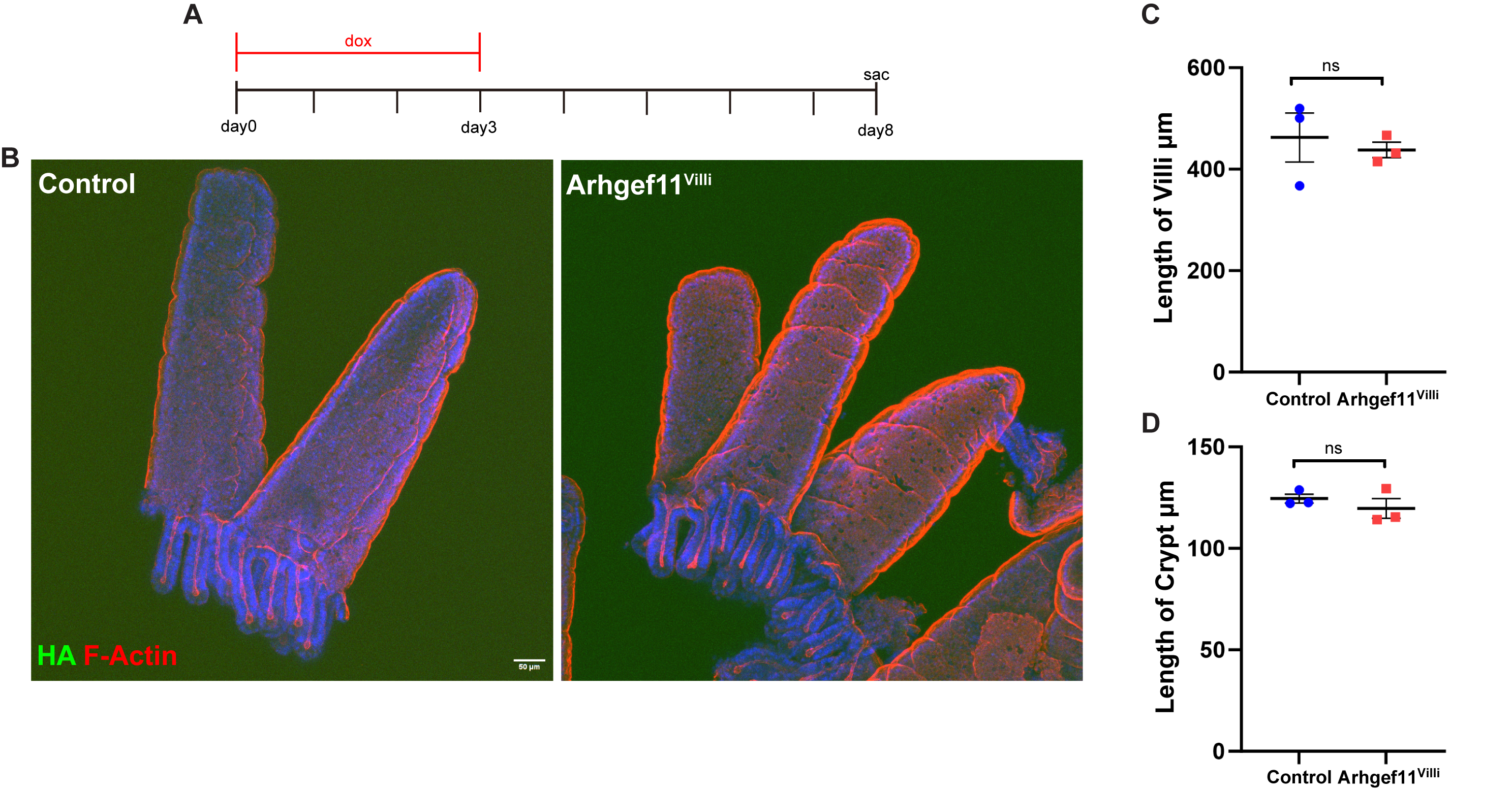

Supplement: S5 Fig — (A)Timeline of experiment. (B) Whole mount immunofluorescent images of control and ArhgefVilli intestinal epithelia stained with HA (green) and phalloidin (red). Scale bar 50μm. (C) Quantification of villi length in microns. For control n = 3 animals (33 villi). For ArhgefVilli, n = 3 animals (43 villi). p = 0.5715, paired t-test. (D) Quantification of crypt length in microns. For control n = 3 animals (109 crypts). For ArhgefVilli, n = 3 animals (134 crypts). p = 0.5710, paired t-test. (TIF) [file pgen.1010899.s005.tif]

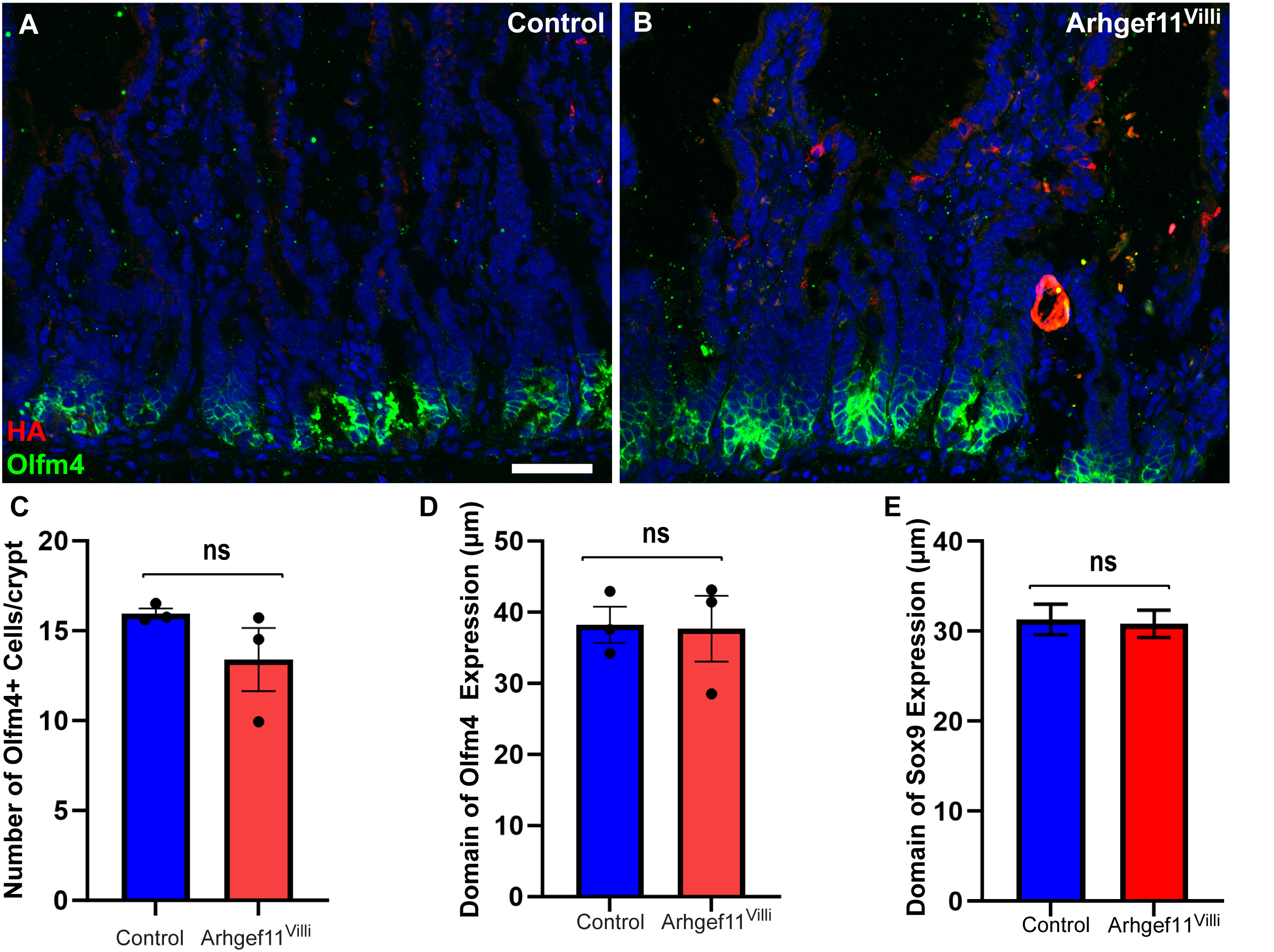

Supplement: S6 Fig — (A-B) Immunofluorescence images of (A) control and (B) ArhgefVilli intestine sections stained with HA (red) and Olfm4 (green) Scale bar 50μm. (C) Number of Olfm4-positive cells per crypt. For control n = 63 crypts from 3 animals. For ArhgefVilli n = 55 crypts from 3 animals. p = 0.2617, paired t-test. (D) Average domain of the expression of Olfm4 in crypt base stem cells. For control n = 78 crypts from 3 animals. For ArhgefVilli n = 63 crypts from 3 animals. p = 0.9190 paired t-test (E) Average domain of the expression of Sox9 in crypt base stem cells. For control n = 62 crypts from 3 animals. For ArhgefVilli, n = 52 crypts from 3 animals. p = 0.8316, unpaired t-test. (TIF) [file pgen.1010899.s006.tif]

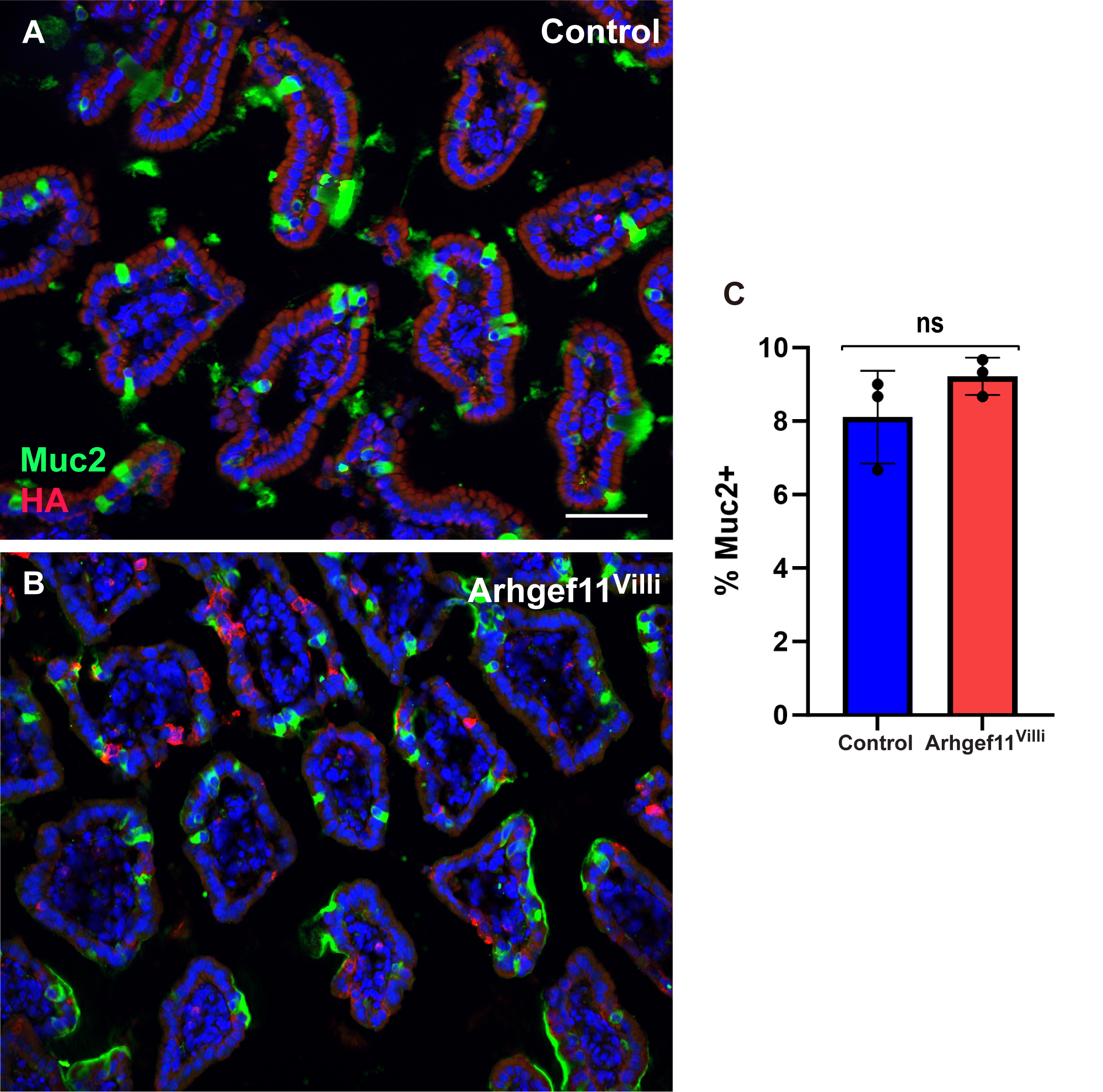

Supplement: S7 Fig — s(A-B) Immunofluorescence staining of Mucin2 (green) and HA (red) in control (A) and Arhgef11Villi (B) villar intestinal epithelia. Scale bar 50 μm. (C) Quantification of the percentage of Mucin2 positive cells out of total villar epithelial cells counted. For control and Arhgef11Villi n = 3 animals (900 cells per animal). p = 0.3624, paired t-test. (TIF) [file pgen.1010899.s007.tif]

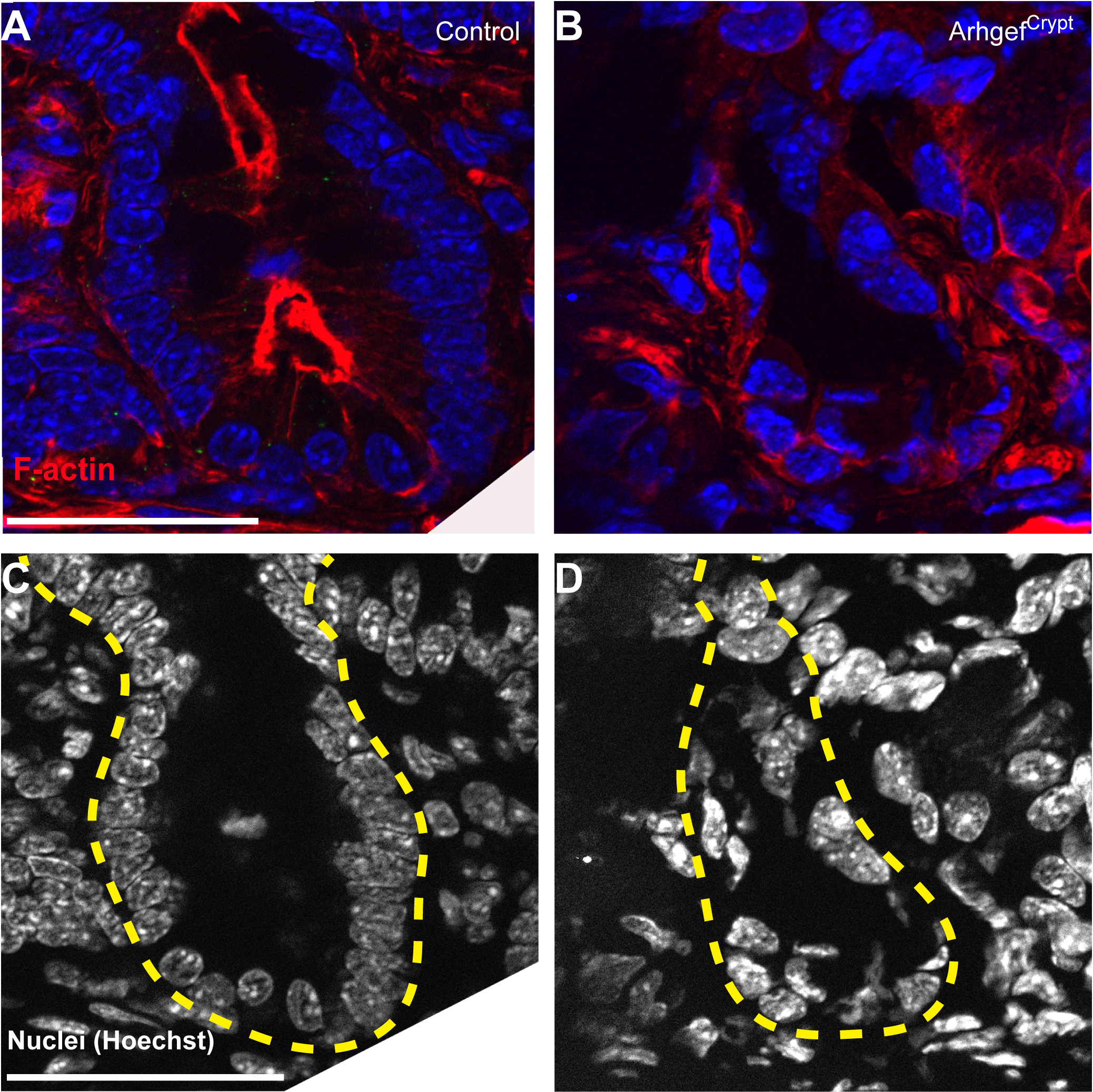

Supplement: S8 Fig — (A-D) Immunofluorescent images of (A,C)control and (B,D)ArhgefCrypt crypt sections stained with (A-B) F-actin (red) or (C-D)Hoechst only (white). Dashed lines denote individual crypts. Scale bar 50μm. (TIF) [file pgen.1010899.s008.tif]
