# Supplementary material for: Compartment specific responses to contractility in the small intestinal epithelium
Source: PLoS Genet. 2024 Mar 22;20(3):e1010899. doi: 10.1371/journal.pgen.1010899 (PMC10990186; doi:10.1371/journal.pgen.1010899)
Supplement: Attachment — Submitted filename: Response To Reviewers.pdf [file pgen.1010899.s010.pdf]

Reviewer 1 comments:

Major comments:

To convincingly show that Arhgef11-CA expression induces contractility in intestinal epithelial cells, the authors should use functional cell, organoid, or explant-based assays (e.g.'s gel contraction/tissue compaction, laser ablation of junctions, genetically encoded tension sensors) as opposed to relying solely on the expression of a few IF markers.

The Arhgef11CA construct has been extensively tested by both us and others in the past to induce contractility in cultured cell lines, primary cells and mice. We have included greater description of this in the text. Optogenetic activation of the construct has been shown to result in rapid contractile responses (Berlew et al 2021). The construct contains only the GEF domain of Arhgef11 and not additional domains that may mediate other functions. Further, when expressed in mouse epidermis (where it is much more uniformly expressed, making these experiments possible), it causes a tissue wide compaction that was measured in ex vivo skin explants (Ning et al, 2021). We have been unable to replicate this in organoids as in this system we lost the compartmentalization of expression and organoids died soon after Arhgef11CA induction. The very similar effects on actin, myosin, cell shape, and junctional tension between the epidermis and the villar epithelium provide strong support for a contractile function.

To convincingly demonstrate that the phenotypes observed are due to cell contractility or tension heterogeneity within the epithelium, the authors should 1) test whether these phenotypes (in both crypt and villus domains) are recapitulated in 2D or 3D intestinal organoids isolated from these mice, and if so whether they can be rescued by ROCK inhibition (e.g. Y27632 or Thiazovivin).

As mentioned above, we are unable to perform these experiments in organoids as we see rapid death after induction of Arhgef11CA in this system (we see widespread expression of the transgene and apoptosis). For this reason, we turned to inhibition of contractility *in vivo* and we chose to assay y-H2aX induction as it is a clear and rapid response to contractility. We fed VillinCre;RosartTA;TreArhgef11 mice doxycycline food, and injected them with 10mg/kg Y27632 (Rock inhibitor) approximately 8 hours later. We then measured the percentage of y-H2aX positive cells per crypt and found that Rock inhibition significantly rescued the DNA damage phenotype in $Arhgef11^{Crypt}$ animals. We have included this quantification in Figure 6 of the manuscript.

They should test whether activation of contractility in vitro through Calyculin A treatment or other means (e.g. lentiviral transduction of other contractility inducers) recapitulates the observed phenotypes.

To address this point, we treated wildtype organoids with Calyculin A and assayed for DNA damage (yH2ax expression). We find that there is a marked induction of yH2aX expression specifically in the crypt base of these organoids after two hours of calyculin a treatment. We have not included this in the manuscript due to the broad effects of Calyculin A.

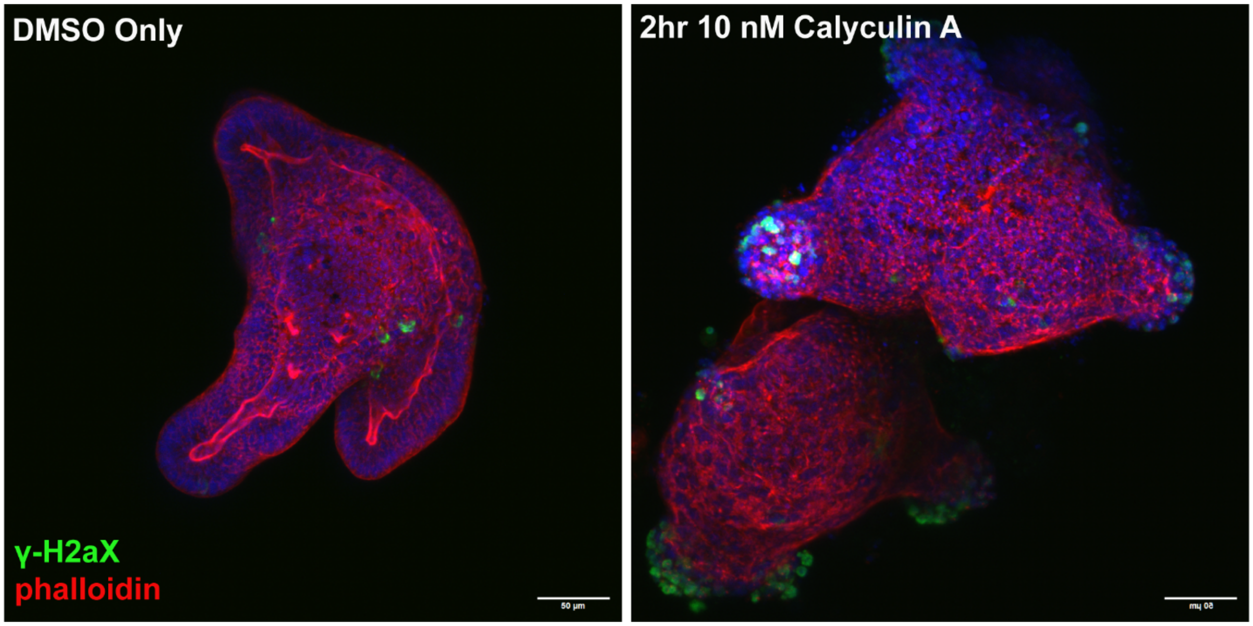

"The changes in contractility appear to be quite local and we have no evidence that they are transmitted through multiple cells." This claim should be investigated in more detail by quantifying the junctional MyoII C etc. as well as phosphorylated myosin light chain in cells adjacent and within the vicinity of the Arhgef11CA-expressing villus cells. From some of the images (e.g. 1I and S2C), it appears that there might be subtle differences that could be more obvious when quantified and would help explain the non-cell-autonomous effects – perhaps one of the most exciting findings of this manuscript.

We included data showing that there is only an increase in α-18 staining in Arhgef11CA-expressing cells (Fig S1C-F). In these quantifications, cells immediately adjacent to Arhgef11CA-expressing cells have α-18 levels that are not significantly different from control tissue.

Minor comments:

The timing of dox induction and tissue collection is unclear for some of the experiments. The manuscript would benefit from adding the experimental timelines to all figures.

We apologize for the confusion and have added diagrams highlighting experimental timelines to necessary figures.

Where along the crypt-villus axis were the line profiles for quantification drawn? Was this specifically for cells along the villus sides or at the tips? The locations could themselves have heterogeneity between them in control samples, so an important control would be to measure the line profiles of Actin/MyoIIC at different positions along the villus or at least clearly state where the measurements are made.

As the primary expression location of Arhgef11-CA expressing cells in the Arhgef11^{Villi} model was the lower third of the villus, our line scan quantifications were consistently measured from this region. We have included this information in the methods section of the text.

Are there changes to villus morphology in the Arhgef11-Crypt mice? It would be assumed that the villi would undergo atrophy but this should be more clearly shown.

There are no major morphological differences in the villus after 24 hours of induction in Arhgef11^{Crypt} mice. Most of the rapid weight loss seems to be due to defects in the colon leading to mis regulation of water absorption. However, after 48 hours of induction, Arhgef11^{Crypt} do begin to exhibit some villus atrophy, consistent with the massive tissue disorganization at that point.

Are the Arhgef11-crypt phenotypes specific to the small intestine, or is the expression/phenotype also observed in the colon?

Quantification of DNA damage in colon samples of Arhgef11^{Crypt} animals suggest that there are similar phenotypes in this tissue as well. However, the induction of Arhgef11CA expression and of DNA damage is more variable from animal to animal in the colon resulting in an upward trend of γ-H2aX expression that does not reach statistical significance via paired t-test. We have included the γ-H2aX colon data in Supplemental Figure 5.

Multiple misspellings of "Arhgef"

Thank you for bringing this to our attention. We have corrected the spelling mistakes in the text.

"Regardless, these data indicate that the crypt hyperplasia, in response to increased actomyosin contractility in villar cells, is driven by transit amplifying cell hyperproliferation and not an expansion of stem cells." This is not convincing from the graph of Sox9 expression alone. To make this claim the authors should look at the proportion of more bona fide ISC markers, such as Lgr5 and Olfm4. Like point #4, the authors need to use a more robust list of markers to support their conclusion that the TA progenitors, not the stem cells, are the cell population driving the hyperproliferative phenotype. Lgr5, Olfm4, Math1, Notch1, etc., markers should be included.

We appreciate this important point. We have now included quantification of Olfm4 expression in control and Arhgef11^{Villi} samples. The length of the Olfm4 expression domain, as well as the number of positive cells per crypt is not significantly different between groups, which is consistent with the Sox9 data. We have added representative images and the quantification to Supplemental Figure 7 and included this data in the text.

"Together these data demonstrate that mechanical deformation induces DNA damage leading to apoptosis of these cells" -Not directly tested that this is causal.

Thank you for this comment. Based on our time course experiments, the events of DNA damage occur before apoptosis (and independently of it), but we did not test whether there is a direct connection. We have adjusted our conclusions in the text to better reflect our observations.

Reviewer 2 comments:

Major comments:

The authors must elaborate on the reasons for actomyosin gain-of-function experiments in the *introduction. It needs to be clarified what was the hypothesis that the authors were trying to test in this study. For example, the following sentence is vague and needs clarification on what function remains unclear: "Despite the importance of actomyosin-mediated contractility in intestinal development and homeostasis, the effects that contractility has on cell and tissue function remain unclear."*

We have expanded on our hypothesis in the text.

"Regulated F-actin dynamics are required for active villar cell migration, driving the homeostatic flux of the tissue [21]. Additionally, actomyosin is required for both cell extrusion, which is important for villar cell turnover, and cell contraction in response to infection[22, 23]. Despite the importance of actomyosin-mediated contractility in intestinal development and homeostasis, and its modulation during pathologic conditions, the effects that contractility has on cell and tissue function remain unclear. This is largely due to the fact that very few tools exist to specifically tune a cell's contractile state *in vivo*, in order to study its cell and tissue level effects. By manipulating contractility *in vivo*, in the absence of additional perturbations, we hope to define the cell and tissue-specific responses that are induced by increased acto-myosin activity."

The authors should refrain from statements that are not accurate, such as: "This study highlights distinct, compartment specific responses to cell contractility and, for the first time, reveals the mechanical sensitivity of small intestinal epithelial stem cells." There are published studies testing "mechanical sensitivity" in the crypt compartment, including stem cells. To name a few: Pentinmikko et al. 2022; Pérez-González et al. 2021; Yang et al. 2021; Sumigray et al. 2018).

· *"for the first time, reveals the mechanical sensitivity of small intestinal epithelial stem cells"*

o Multiple studies, included some cited here, have shown this (e.g. PMIDs: 31019299, 31019299, 31019299, 31019299)

We apologize that this statement was not as clear as intended. We have adjusted it to state that they are highly sensitive to internal mechanical forces generated by the acto-myosin cytoskeleton. Previous work focused on external forces.

The authors should explore the expression of NMIIA, which is also expressed in the intestinal epithelium, and they are strongly encouraged to stain for pMLC, which indicates activated myosin and contracting stress fibers.

We have included images and quantification MyoIIA staining in Supplemental Figure 1, which is also increased at lateral junctions in Arhgef11-CA positive cells. While we have stained for pMLC in both Arhgef11^{Villi} and Arhgef11^{Crypt} models , the panel of antibodies we have tried yielded inconsistent results, including in areas where pMLC is known to be active, such as the muscle wall of the small intestine.

A more robust panel of cell polarity markers needs to be assessed to determine changes in cell polarity.

We have added ZO-1 (apical junction/tight junction maker), E-cadherin (lateral membrane marker), and pericentrin (centrosome, which localizes apically) to our panel of cell polarity markers in Supplemental Figure 2. All showed a normal localization.

An analysis of cell death in villi is needed. Even though the authors show that villus length is maintained in mutant mice, the increase in crypt proliferation might result in proper villus length by replacing lost villus cells. Their conclusion that the only observed phenotype in villus cells is the change in morphology will be further supported if there's no difference in cell death in villus cells.

We have analyzed cell death in the Arhgef11^{Villi} model, and do not see any HA+ cells with cleaved caspase-3 staining. Further, while it is difficult to find apoptotic cells being extruded at the tips of villi, our observations of mutant and control tissue do not suggest there is an increase in apoptotic cells being extruded from the tips, or any other region of the villus.

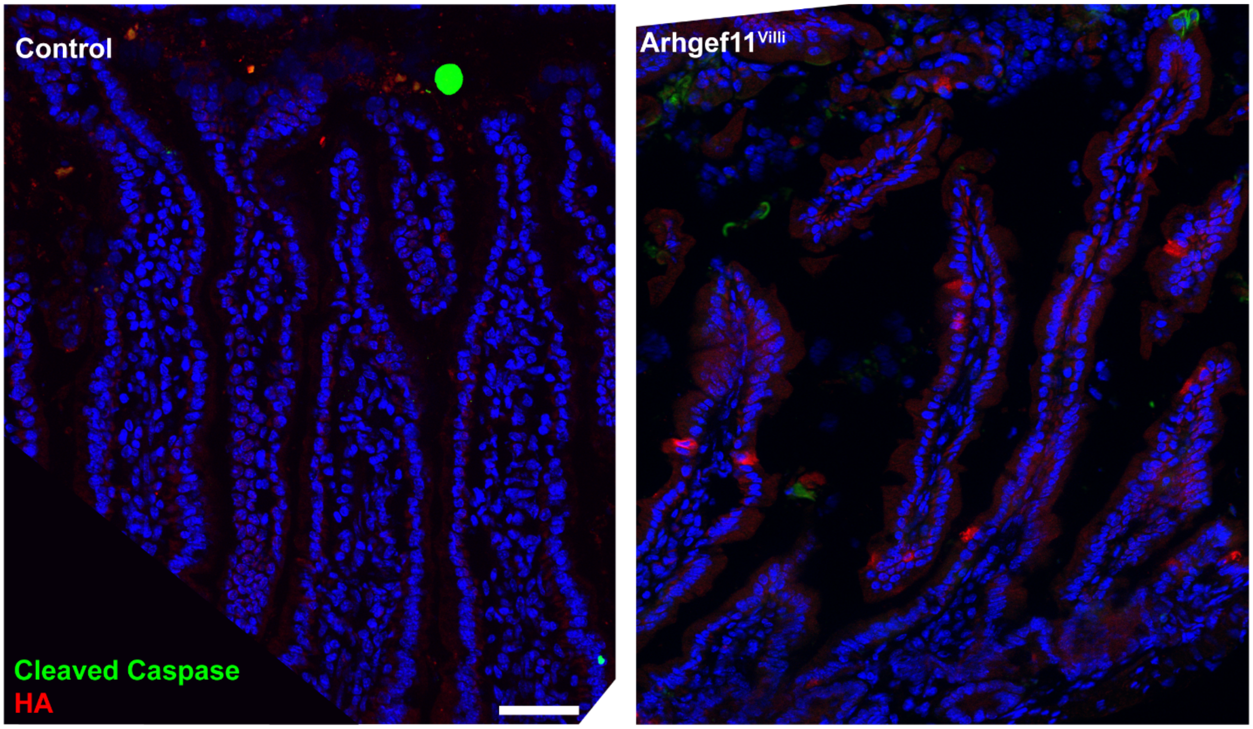

The authors cannot conclude that Arhgef11 gain-of-function is restricted uniquely to villus cells since they show the presence of HA+ cells high up in the crypt compartment. Additionally, the authors' conclusion

that there is a non-cell autonomous effect from villus cell contraction cascading to the proliferative compartment is intriguing and relevant. Still, it is not supported by their current data. Their manuscript and conclusion will require a villus-specific driver, for example, K20CreER, to drive the expression of their allele specifically and uniquely in differentiated villus cells.

There is only a very small number of crypt cells expressing Arhgef11-CA in the Arhgef11^{Villi} model (0.5% of all crypt cells counted; only 7% of crypts had any positive cells; 147 total crypts from 4 animals), making it very unlikely that effects are due to these cells (especially as expression in crypt cells results in apoptosis). We have used a more representative image of the transgene expression in Arhgef11^{Villi} tissue (Figure 1) and have also improved the accuracy of the axis label on Figure 1C.

We attempted to improve on the specificity of this already quite specific expression using a MafB-Cre line (Jax Strain #:029664), which we have previously shown to drive enterocyte-specific expression (Bara et al., 2022). We found that the Rosa-lox-Stop-lox-rtTA line we used had leaky expression specifically in crypt cells precluding us from doing the analysis.

Are there any defects in cell differentiation after Dox induction?

We stained for MafB and find no defects in enterocyte expression in Arhgef11^{Villi}. We also stained for Mucin2 and find no significant difference in the number of goblet cells in control and mutant tissue. We included the Mucin2 data in Supplemental Figure 3.

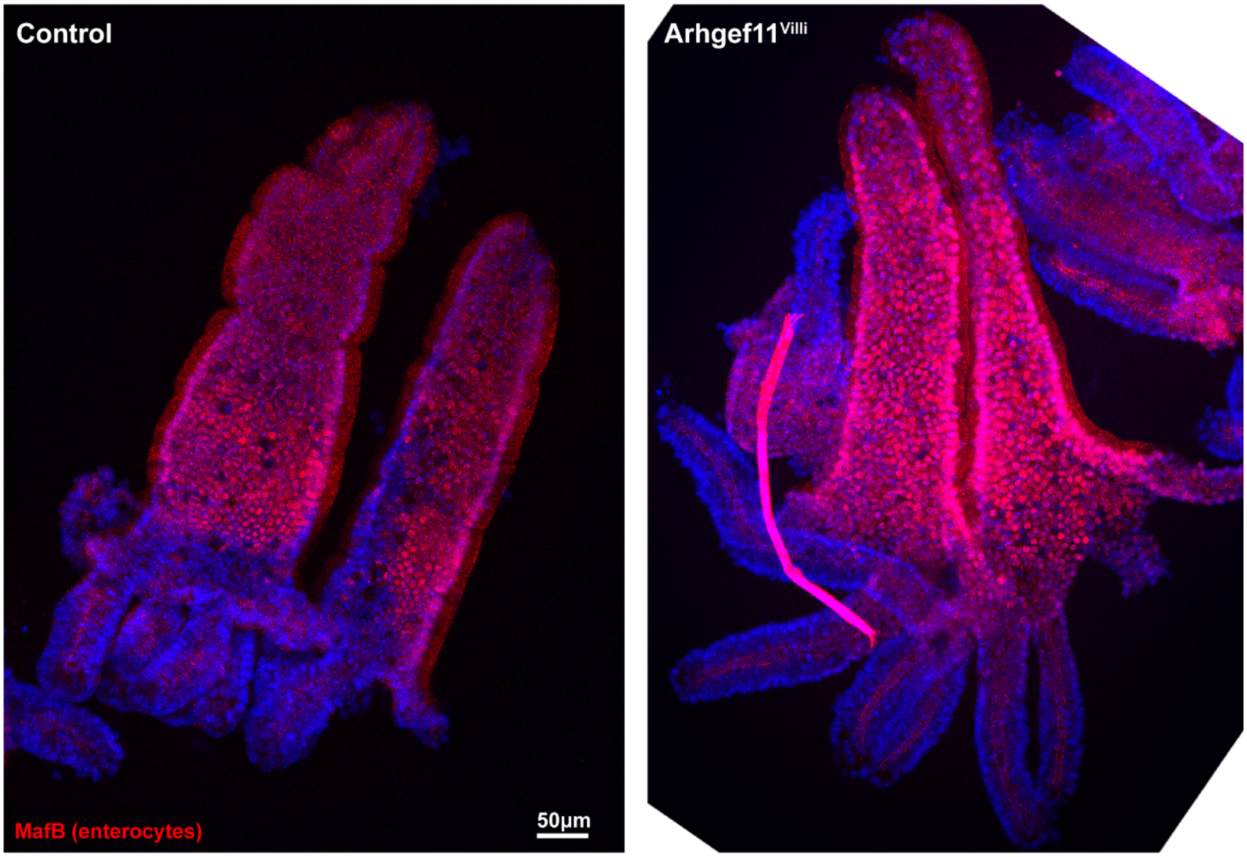

The authors claim that recombination in the Arhgef11Villi mouse model occurs only in villus cells and cells at the villus-crypt junction, which are differentiated cells lost within 3-5 days in the small intestine. Thus, is the phenotype reversible? After the mosaic recombined differentiated cells are replenished by new non-recombined cells, do the villus and crypt phenotypes back to homeostasis?

Thank you for this suggestion. We find that the crypt hyperplasia phenotype is reversible five-days after ceasing transgene expression induction. This data is in Supp Fig 6.

Have the authors tested increasing the Dox induction paradigm of the Arhgef11Villi mouse model for longer than 3 days? This is an interesting experiment to carry out to support their conclusion that the crypt phenotype is driven by TA cells. As TAs don't self-renew, a likely outcome would be that these cells exhaust themselves, and the crypt phenotype should resolve back to homeostasis.

We have tested out to five days of transgene expression. The crypt did not return to its previous length, however, it appeared to reach a new stead state with lengths comparable to three days of treatment.

The authors should adjust their conclusions to reflect their data. For the authors to conclude that "crypt progenitor cells are more sensitive to increased actomyosin contractility" they need to induce actomyosin contractility in the progenitor cells. Their current Arhgef11crypt genetic tool is active throughout the crypt. Thus, the role of the distinct crypt cell populations (stem cells, Paneth cells, secretory progenitors, and absorptive progenitors) cannot be disentangled.

Progenitor cells refers to both stem cell and transit amplifying cells. We do not distinguish between them here. However, based on the pattern of staining of DNA damage and apoptosis, it appears that both of these populations are likely affected. Due to species cross-reactivity of our antibodies we are unable to stain for both DNA damage and Olfm1 at the same time.

Is the basement membrane of the injured crypts impaired?

To address this, we stained with Collagen IV in Control and Arhgef11^{Crypt} tissue to visualize the organization of the basement membrane. However, we find no apparent differences in collagen IV intensity or organization in the disorganized crypts, at least at the early time points examined. We have not included this data in the manuscript.

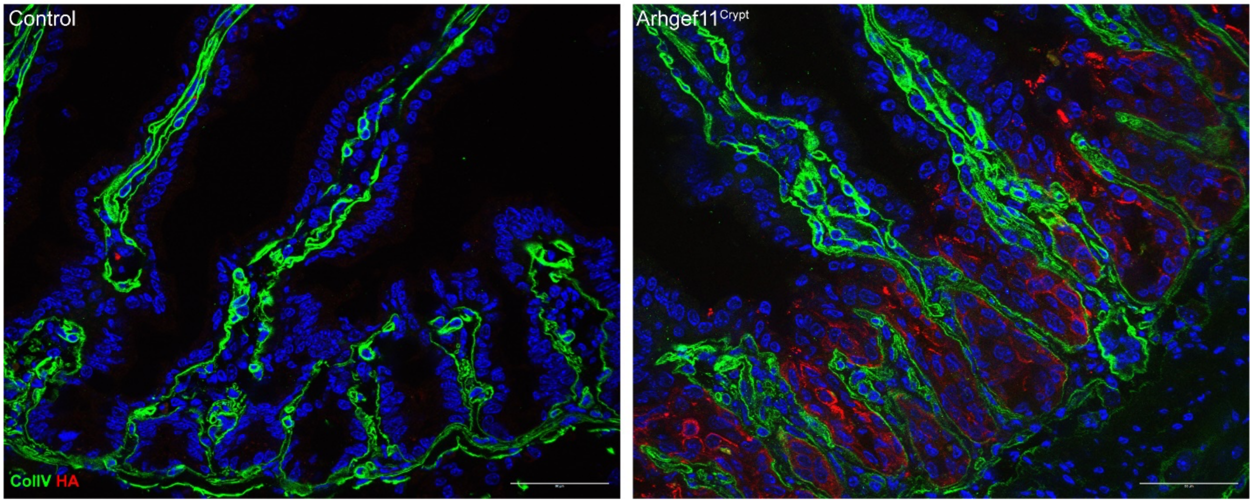

Other comments:

Statistics should be done on the average "measured unit" per mouse and not using each data point. For example, the statistical analysis of Fig. 2C-F needs to be reanalyzed using the average villi length –for C– or the average crypt length –for F– per mouse instead of doing the statistics using each villus (n=20-40) or each crypt (n=51-64) data point. The correct statistical analysis that should be applied across the manuscript is illustrated in Fig. 2H. Related to point #9. The authors are encouraged to show the distribution of the average "measured unit" per mouse as individual values using graphs such as Fig. 2H and avoid using bar graphs that do not show the individual values, such as Fig. 2I.

We have remade all relevant graphs to reflect each mouse as a data point. We have also re-run the statistical analyses as paired t-tests.

Can the authors choose two representative images in Fig. 4I instead of the current low-magnification image, which does not get their point across?

Thank you for bringing this to our attention. We have removed the images that are difficult to interpret and left only the graph with the length measurements for clarity.

In Fig. 4D-E, the authors should add a reference point on where these images are taken along the crypt axis, i.e., are the cross sections at the +4 position, mid-crypt, or below the crypt-villus hinge? This information would be helpful since the lumen of the crypt changes in size as you move up the crypt axis. Further, these panels will significantly improve if the authors use their whole mount tissue for 3D reconstruction of the control and mutant crypts.

We apologize for the confusion. Our quantifications were always taken at the mid-point of the crypt, as this is the region where the apical lumen of the crypt cells is smallest. We have made this clarification in

the text, as well as in Fig 4D-E. Additionally, we added a sagittal plane image to highlight the fact that the cryptal lumen is fairly uniform throughout the length of the crypt in control tissue.

It needs to be clarified what section of the small intestine was used for their analysis. In the methods section, they mentioned the “medial” section of the small intestine. Does this mean Jejunum? Either use the duodenum, jejunum, or Ileum terminology or clarify how far from the pylorus or pyloric sphincter the analysis was made. This is relevant because of the transcriptional, cellular, and morphological differences along the proximal-distal small intestine.

We have adjusted to state that we focused on the jejunum.
