# Supplementary material for: Compartment specific responses to contractility in the small intestinal epithelium
Source: PLoS Genet. 2024 Mar 22;20(3):e1010899. doi: 10.1371/journal.pgen.1010899 (PMC10990186; doi:10.1371/journal.pgen.1010899)
Supplement: Attachment — Submitted filename: 2ndrevision_response.pdf [file pgen.1010899.s013.pdf]

Response to Reviewers

Reviewer #1

In this revised manuscript, Hinnant et al. performed additional experiments, quantifications, and clarifications to address my concerns in the initial submission, including an *in vivo* rescue of some aspects of the phenotype with ROCK inhibition. They have adequately provided justification for use of the Arhgef11 allele and explained why an in vitro system to directly probe some of these cellular mechanisms is unachievable with current technologies (e.g. rapid death of intestinal organoids). This version of the manuscript clearly shows that there are distinct responses to enhanced epithelial contractility in villi and crypts and paves the way for further mechanistic studies. I have a few minor suggestions prior to publication:

- For transparency to readers, I encourage the authors to include a sentence describing their finding that Arhgef11 induction causes a loss of tissue compartmentalization and death in organoids. I also find this result intriguing because it further supports the notion that an optimal degree of epithelial contractility is important for homeostasis.

We have included a sentence in the Discussion stating this finding.

- The HA signal is difficult to see in Fig. 1B compared to the version in the original submission and should be made brighter (or a better example image should be used).

We have added a new set of representative images with clearer staining to Figure 1B.

- Text should read epithelium instead of epidermis in sentence: "…where they appear to have been extruded from the **epidermis**…"

Thank you for catching this error. It has been corrected.

Reviewer #2

Unresolved comments:

- Would the authors address this comment –initially included in the revision document as point #7– that was not added in their response to reviewers?

  Like point #4, the authors need to use a more robust list of markers to support their conclusion that the TA progenitors, not the stem cells, are the cell population driving the hyperproliferative phenotype. Lgr5, Olfm4, Math1, Notch1, etc., markers should be included.

  "Regardless, these data indicate that the crypt hyperplasia, in response to increased actomyosin contractility in villar cells, is driven by transit amplifying cell hyperproliferation and not an expansion of stem cells." This is not convincing from the graph of Sox9 expression alone. To make this claim the authors should look at the proportion of more bona fide ISC markers, such as Lgr5 and Olfm4. Like point #4, the authors need to use a more robust list of markers to support their conclusion that the TA progenitors, not the stem cells, are the cell population driving the hyperproliferative phenotype. Lgr5, Olfm4, Math1, Notch1, etc., markers should be included.

We have addressed this point in the initial revision. Both reviewers asked for this, and we mistakenly only included the response to the first reviewer. See our initial response below:

We appreciate this important point. We have now included quantification of Olfm4 expression in control and Arhgef11^{Villi} samples. The length of the Olfm4 expression domain, as well as the number of positive cells per crypt is not significantly different between groups, which is consistent with the Sox9 data. We have added representative images and the quantification to Supplemental Figure 7 and included this data in the text.

- The authors need to expand on their response to point #12.

    Authors response to point #12:
    Progenitor cells refers to both stem cell and transit amplifying cells. We do not distinguish between them here. However, based on the pattern of staining of DNA damage and apoptosis, it appears that both of these populations are likely affected. Due to species cross-reactivity of our antibodies we are unable to stain for both DNA damage and Olfm1 at the same time.

    1st: The authors are strongly encouraged to distinguish between stem cells and TAs and avoid using “progenitor cells” to refer to both cell populations in the intestine (i.e. stem cells+TAs). This should be reflected throughout the text.

Where appropriate, and when clear, we try to use the terms stem and TA cell. However, occasionally it is useful to refer to all the proliferative cells in the crypt (especially when they are behaving similarly) collectively as progenitors.

    2nd: The manuscript would greatly benefit from showing which crypt cell population is undergoing cell death. The authors can bypass the issue of species cross-reactivity of their antibodies by using TUNEL staining with any antibody they choose for stem cells and TAs. Another alternative is to use their DNA damage or CC3 antibody with RNAscope probes specific to mRNA expressed in stem cells and TAs.

We have included a new panel in Figure 6 that shows both Olfm+ve crypt base cells, and Olfm-ve upper crypt cells can both be marked by γ-H2aX. This demonstrates that both stem and transit amplifying cells respond similarly to increased contractility. Additionally, we note that caspase positive cells were also found at both crypt base and in the upper crypt, again supporting that idea that both TA and stem cells are dying.
