# Supplementary material for: Compartment specific responses to contractility in the small intestinal epithelium
Source: PLoS Genet. 2024 Mar 22;20(3):e1010899. doi: 10.1371/journal.pgen.1010899 (PMC10990186; doi:10.1371/journal.pgen.1010899)
Supplement: Attachment — Submitted filename: Hinnat et al._PLOS Genetics_2nd revisions.docx [file pgen.1010899.s011.docx]

**Compartment specific responses to contractility in the small intestinal epithelium**

Unresolved comments:

- Would the authors address this comment –initially included in the revision document as point #7– that was not added in their response to reviewers?

Like point #4, the authors need to use a more robust list of markers to support their conclusion that the TA progenitors, not the stem cells, are the cell population driving the hyperproliferative phenotype. Lgr5, Olfm4, Math1, Notch1, etc., markers should be included.

- The authors need to expand on their response to point #12.

Authors response to point #12:

Progenitor cells refers to both stem cell and transit amplifying cells. We do not distinguish between them here. However, based on the pattern of staining of DNA damage and apoptosis, it appears that both of these populations are likely affected. Due to species cross-reactivity of our antibodies we are unable to stain for both DNA damage and Olfm1 at the same time.

1^st^: The authors are strongly encouraged to distinguish between stem cells and TAs and avoid using “progenitor cells” to refer to both cell populations in the intestine (i.e. stem cells+TAs). This should be reflected throughout the text.

2^nd^: The manuscript would greatly benefit from showing which crypt cell population is undergoing cell death. The authors can bypass the issue of species cross-reactivity of their antibodies by using TUNEL staining with any antibody they choose for stem cells and TAs. Another alternative is to use their DNA damage or CC3 antibody with RNAscope probes specific to mRNA expressed in stem cells and TAs.
