# Supplementary material for: Compartment specific responses to contractility in the small intestinal epithelium
Source: PLoS Genet. 2024 Mar 22;20(3):e1010899. doi: 10.1371/journal.pgen.1010899 (PMC10990186; doi:10.1371/journal.pgen.1010899)
Supplement: Attachment — Submitted filename: Hinnant Lechler Revisions.docx [file pgen.1010899.s012.docx]

In this revised manuscript, Hinnant et al. performed additional experiments, quantifications, and clarifications to address my concerns in the initial submission, including an *in vivo* rescue of some aspects of the phenotype with ROCK inhibition. They have adequately provided justification for use of the Arhgef11 allele and explained why an in vitro system to directly probe some of these cellular mechanisms is unachievable with current technologies (e.g. rapid death of intestinal organoids). This version of the manuscript clearly shows that there are distinct responses to enhanced epithelial contractility in villi and crypts and paves the way for further mechanistic studies. I have a few minor suggestions prior to publication:

- For transparency to readers, I encourage the authors to include a sentence describing their finding that Arhgef11 induction causes a loss of tissue compartmentalization and death in organoids. I also find this result intriguing because it further supports the notion that an optimal degree of epithelial contractility is important for homeostasis.
- The HA signal is difficult to see in Fig. 1B compared to the version in the original submission and should be made brighter (or a better example image should be used).
- Text should read epithelium instead of epidermis in sentence: “…where they appear to have been extruded from the **epidermis**…”
